# 232. Safety and Effectiveness of Intravenous to Oral De-escalation Compared to Continued Vancomycin Therapy in Orthopedic Infections

**DOI:** 10.1093/ofid/ofab466.434

**Published:** 2021-12-04

**Authors:** Chanah Gallagher, Russell J Benefield, Laura Certain

**Affiliations:** 1 University of Utah, Salt Lake City, Utah; 2 University of Utah Health, Salt Lake City, UT

## Abstract

**Background:**

The Oral versus Intravenous Antibiotics for Bone and Joint Infection (OVIVA) trial determined oral antibiotics administered during the first six weeks of therapy were non-inferior to parenteral antibiotics. There was no difference in the incidence of serious adverse effects. The objective of this study was to evaluate the safety and effectiveness of de-escalating to oral therapy compared to continuing parenteral vancomycin therapy in patients with orthopedic infections in a real-world setting.

**Methods:**

We conducted a single-center, retrospective cohort study of patients discharged between April 1, 2018 and April 1, 2020 with an orthopedic infection, a prescription for at least four weeks of parenteral vancomycin, and documented follow-up. The primary outcome was incidence of adverse events defined as provider documentation of the event and changes to therapy. The secondary outcome was incidence of 6-month treatment failure defined as repeat surgical intervention or therapy escalation.

**Results:**

One hundred fifty-seven patients were included. Twenty-nine (18.5%) patients were de-escalated to oral therapy. Three (10%) patients in the oral therapy group had an adverse event compared to 35 (27%) in the vancomycin group (p=0.058). Of the 35 patients with an adverse event in the vancomycin group, eight were due to parenteral access-related complications. Treatment failure occurred in three (10%) patients in the oral therapy group compared to 27 (21%) patients in the vancomycin group (p=0.29). Three (10%) patients in the oral therapy group had an unplanned readmission compared to 25 (20%) patients in the vancomycin group (p=0.24).

Baseline Characteristics, Unplanned Readmission Rates, and Incidence of Adverse Events and 6-Month Treatment Failure

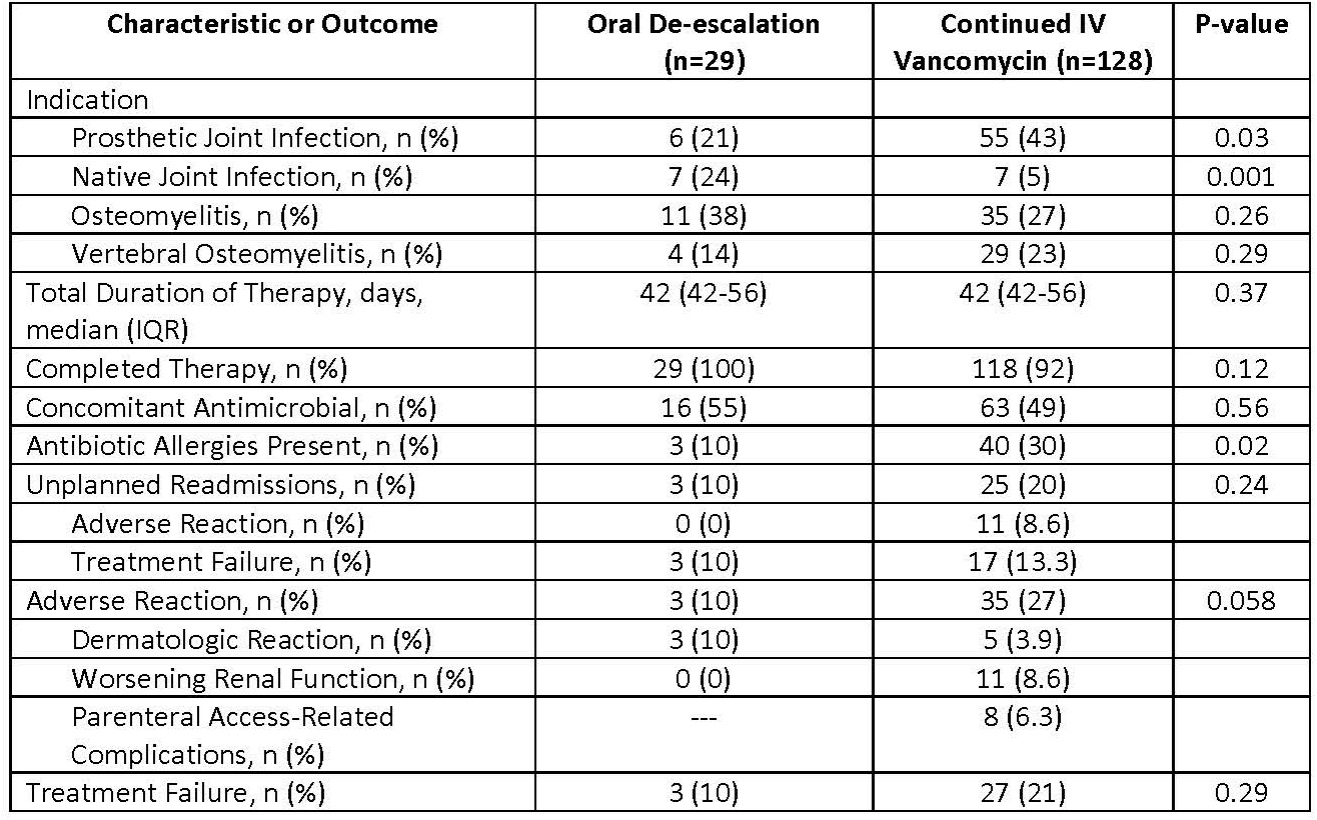

**Conclusion:**

Patients de-escalated to oral therapy had fewer adverse events and similar incidences of treatment failure compared to patients maintained on parenteral vancomycin. Switching to oral therapy avoids some adverse events related to parenteral access. Our results in an uncontrolled, real-world setting are consistent with the OVIVA trial. Though limited by sample size, our data indicate switching to oral therapy in patients with an orthopedic infection improves safety outcomes without compromising effectiveness compared to continued parenteral vancomycin therapy.

**Disclosures:**

**Russell J. Benefield, PharmD**, **Paratek Pharmaceuticals** (Grant/Research Support)

